# Gambling Despite Nationwide Self-Exclusion–A Survey in Online Gamblers in Sweden

**DOI:** 10.3389/fpsyt.2020.599967

**Published:** 2020-12-02

**Authors:** Anders Håkansson, Carolina Widinghoff

**Affiliations:** ^1^Faculty of Medicine, Department of Clinical Sciences Lund, Psychiatry, Lund University, Lund, Sweden; ^2^Malmö Addiction Center, Region Skåne, Malmö, Sweden

**Keywords:** gambling disorder, problem gambling, online gambling, online casino, behavioral addiction, self-exclusion, harm reduction

## Abstract

**Background:** Voluntary self-exclusion is a well-known harm reduction intervention in problem gambling, although primarily in operator-specific or venue-based systems. A nationwide overall self-exclusion system (“*Spelpaus”*) for all licensed gambling was introduced in Sweden in 2019. However, gambling in overseas companies despite national exclusion may be a concern in online gamblers. The present web survey study aimed to study self-reported self-exclusion and gambling despite exclusion in a nationwide multi-operator land-based/online exclusion system.

**Methods:** Web survey in web panel members of a market survey company, carried out in May, 2020 (co-occurring with the COVID-19 pandemic). Past-year online gamblers (*n* = 997) answered questions about gambling patterns, gambling problems, psychological distress, self-exclusion since “*Spelpaus”* introduction, and gambling despite self-exclusion.

**Results:** Seven percent reported ever self-excluded at *Spelpaus*, and this was associated with younger age, female gender, gambling problems, and chance-based games and online poker. In logistic regression, *Spelpaus* remained strongly associated with past-year online casino gambling, gambling problems, and absence of past-year sports betting. Among those having self-excluded, 38 percent reported gambling despite self-exclusion, most commonly online casino.

**Conclusions:** In online gamblers in a setting with a nationwide self-exclusion system, using this was associated with past-year online casino gambling and gambling problems. Gambling despite self-exclusion appears to be common, and more commonly involves online casino. Stakeholders should aim to increase rates of self-exclusion in high-risk online gamblers, both during and beyond the COVID-19 situation in which the study was carried out. Also, policy makers should use gambling regulation in order to decrease the risk of breaching self-exclusion online, such as through the prohibition of non-registered gambling operators. Further research should focus on in-depth analysis of the reasons for gamblers to enroll or not enroll in multi-operator self-exclusion.

## Introduction

Problem gambling is a condition known to have severe consequences on the mental well-being, social and financial situation of affected individuals, and has been reported to affect somewhere between less than one percent and almost six percent, across different studies and settings (1). Gambling disorder (2) is a criteria-based diagnosis recognized by the World Health Organization diagnostic system, ICD-11 (3), and the American Psychiatric Association's diagnostic manual (4), nowadays as one of the addictive disorders along with drug and alcohol use disorders. Gambling disorder is associated with a high degree of psychiatric comorbidity (5, 6), and typically severe financial difficulties (7).

Although there is growing scientific support in favor of treatment of gambling disorder, such as through cognitive-behavioral therapy (8) or brief (9) or motivational interventions (10), treatment seeking is known to be low and associated with different barriers (11). Besides formal treatment, and in particular for individuals with problem gambling even in the absence of formal treatment seeking, voluntary self-exclusion from gambling is a commonly used harm reduction instrument (12, 13). Such self-exclusion tools, however, have been scientifically studied in several land-based gambling settings (14–19), meaning that an individual self-excludes from entering one or several specific gambling venues, such as land-based casinos. Also, there are reports about self-exclusion tools on specific online gambling sites (20–22), i.e., where a gambler self-excludes from one specific gambling operator.

In recent years, online gambling plays an increasing role in gambling markets and in the patient population of individuals with gambling problems. Online gambling may present a number of particular hazards to the gambling population, mainly due to the characteristics of the online modality in itself, being rapid and highly accessible (23) to an extent which is difficult to compare to any land-based venues. In some settings, such as the one studied here, online gambling represents a very large proportion of treatment-seeking patients (24). Online gambling is known to be highly predominating in individuals with high-risk gambling in the present setting, and recent data have indicated that this may also confer changing gender patterns, with the percentage of women becoming larger in populations with gambling problems (25, 26). While a majority of people reporting self-exclusion are typically male (27, 28), as are typically a majority of individuals reporting problem gambling in most settings (1), there is so far less knowledge about the gender distribution in nationwide multi-operator self-exclusion services.

Online gambling presents particular challenges to gamblers who want to self-exclude from a problematic gambling behavior; gambling operators online are numerous, and the self-exclusion from one site may easily be followed by the registration and gambling on another site in order to enable continued gambling. Also, it has been shown that the risk of relapsing into gambling in other sites than the one excluded from is perceived as a major limitation to this method (27). Sweden, after a major change in the gambling market legislation from January 1st, 2019, has introduced a nationwide self-exclusion system from all types of licensed gambling types in the country, and administered by a government authority (29). Despite the theoretically broad coverage of such a system, there is limited knowledge about the extent to which overseas gambling and other non-regulated gambling opportunities may limit the performance of this self-exclusion system. A recent web survey from the present setting demonstrated that—unsurprisingly—respondents with problem gambling were more likely than the remaining respondents (who were not actively gambling or gambled but screened negative for problem gambling) to enroll in such a self-exclusion system (30). However, little is known about how such self-exclusion is influenced by the risk of gambling on gambling services not covered by the system, a theoretical risk particularly in settings with widespread online gambling opportunities.

As there is little research in the area of nationwide multi-operator self-exclusion from gambling, and given the particular challenges of online gambling, the present study aimed to increase knowledge about which online gamblers enroll in such a system, and about the risk of online gamblers breaching it. The present analysis uses a database of online gamblers assessed in a web survey in May, 2020, in order to study online behavior, problem gambling, indebtedness and self-exclusion. From this database, one prior study has been published (31), using the fact that the data were collected during the COVID-19 pandemic, and studying potential pandemic-related effects on gambling. Using the same population of past-year repeated-occasion online gamblers, the present analysis aimed to study the use of self-exclusion in a setting with an overall, combined land-based/online and multi-operator self-exclusion service. Specifically, the study aimed to assess, in online gamblers, variables associated with having self-excluded, such as specific gambling patterns, psychological distress, gender, age and living conditions, as well as to study potential gambling despite self-exclusion and correlates of such self-exclusion breaching.

## Methods

The present study is a web survey addressing online gamblers in Sweden, recruiting from members of a pre-existing web panel of the market survey company Ipsos. Members of the web panel regularly receive offers to participate in market surveys and political opinion polls, and the company also has carried out research studies within their web panel, such as in the area of research reported here (32, 33). In a previous gambling-related study using the same web panel, participants were seen to be skewed toward higher level of education and higher monthly income, compared to the general Swedish population (33).

The present project was reviewed by the Swedish Ethical Review Authority (file number 2020-00364), which expressed no ethical concerns with the project and stated that it formally did not require ethical approval as it does not include personal data possible to link to an identified individual.

### Setting

Since January 1, 2019, a national self-exclusion instrument for gambling, *Spelpaus* (www.spelpaus.se) is in use in Sweden, as part of a new gambling market legislation (29). An individual, with or without a current gambling behavior, can register voluntarily using an official online identification service and provided she/he is above 18 years of age (legal gambling age in Sweden), and is thereafter self-excluded for a period of the individual's own choice; 1, 3, 6 months, or for unlimited time but with the possibility of discontinuation after 12 months. One self-exclusion period can immediately be actively followed by another one, and the administration of this system does not require any registration or visit to a gambling operator's site. Upon every gambling occasion of an individual at any gambling licensed gambling site, an electronic control is made with the national *Spelpaus* register, such that an individual can be allowed to gamble only provided she/he is not currently self-excluded. Until now, around 50,000 individuals have so far self-excluded using this service, corresponding to slightly above half a percent of the adult population in Sweden. About 75 percent of these individuals who have self-excluded are reported to be men (34).

The *Spelpaus* system applies to licensed operators, which include the state-owned gambling operator AB Svenska Spel (providing sports betting, online poker, land-based electronic gambling machines, online bingo, online lotteries, and online casino games), the state-owned land-based casinos (four in total in Sweden, owned by a sub-division of AB Svenska Spel), and a large number of operators offering online casino games, sports or horse betting, online bingo, online poker, and online lotteries. Gambling types not included in the self-exclusion system include land-based lotteries such as lottery tickets bought in coffee shops, gas stations, grocery stores and similar, and so-called “restaurant casinos,” which refer to smaller dealer-administered gambling services provided in bars and restaurants and limited to the deposit of smaller amounts.

### Procedure

The present study applied the same recruitment method and the same criteria of inclusion as one previous study in online gamblers, carried out in the present setting in 2018 (33). The study was conducted from May 5 to May 12, 2020. Thus, the present study was conducted during the ongoing COVID-19 pandemic and the restrictions to society surrounding it, a situation recently highlighted as potentially affecting online gambling behavior (29). For example, a fear of a potential increase in some gambling types, typically online gambling, has been discussed, particularly during periods of lock-down of land-based gambling venues and sports events (35, 36). In addition to the purposes of the original project, the fact that is was carried out during the COVID-19 pandemic gave rise to a first publication, where past-30-day gambling was assessed as a measure of gambling habits during the pandemic (31). Both in that study and in another general population survey in Sweden, possible decreases have been seen in self-report data for a number of gambling types, such that more individuals in a survey study reported a decrease in gambling during the pandemic, compared to those reporting an increase (35). In the analyses of the present paper, past-30-day gambling habits were not assessed specifically, but instead, individuals were included because of reporting online gambling on ten occasions or more during the past year, and the gambling variables assessed were the full measure of having gambling on a particular gambling type at any time, either during the past 30 days, or during the past year prior to that.

Web panel members were asked about how many times during the past year they had gambled on online betting or online casinos, and respondents endorsing the option of 10 times or more, were further assessed in the study. The invitation to participate included written online information about the study, and informed consent was needed in order to open the survey. Participation in the web survey renders a monetary compensation in the form of credit points in the market survey company's own credit system, where the participation in the present kind of study provides credits of a value of around 1.50 Euros. The aim of the study was to include 1,000 individuals, and when inclusion was halted, 1,007 responses had been registered. For 10 individuals, data on gambling problem severity were missing, such that a total of 997 individuals were included in the final sample.

### Measures

Self-exclusion was assessed with a brief introducing sentence about the new national system in used since January, 2019, and asked whether the respondent had ever—since the start of that system—used it for self-exclusion. If yes, the next question asked about the period of time chosen (1, 3, 6, or 12 months). Thereafter, questions were asked about whether the respondent had had any gambling of other types during the self-exclusion, and for each of the gambling types included, whether that had been gambled or not during the self-exclusion period.

Among those endorsing the self-exclusion item, one individual reported among “other” games gambled during self-exclusion that she/he mistakenly had chosen the “yes” option, and stated in free text that she/he had not self-excluded.

Problem Gambling Severity Index [PGSI, (37)] was used for the assessment of problem gambling, as in the previous study in online gamblers (33) and in other general population research from the present setting (25). As in previous studies, 0 point was regarded as no-risk gambling, 1–2 points as low-risk gambling, 3–7 points as moderate-risk gambling, and 8 points or above as problem gambling (25, 33). Other data used in the present study include gender, age (in age groups), living conditions (with several options which were *post-hoc* dichotomized as either living alone without children, or living with somebody), occupation (several options, dichotomized as either working/studying, or not), whether the respondent had ever felt a need to seek treatment for problem gambling, and questions about psychological distress. The measure of psychological distress was the Kessler-6 scale (38), consisting of six items describing mental health symptoms and scored 0–4 for each item, summarized to a total score of 0–24. The Kessler-6 scale assesses the past 6 months, and has been validated as a good measure of psychological distress (39, 40). In the present study, a total score of five or more was considered to represent psychological distress on at least a moderate level.

Gambling habits were assessed with questions about any gambling during the past 30 days for each of the gambling types displayed in Table 1, and for individuals denying each of the gambling types, the next question was asked about whether the individual had gambling on that form of gambling during the past year but prior to the last 30-day period. Here, gambling was reported as any past-year gambling, i.e., the endorsing of any of these two questions for each form of gambling. For sports betting, in the statistical analyses here, both sports live betting and non-live betting were collapsed.

**Table 1:** Characteristics of the study sample (*N* = 997).

Male gender	75% (744)
**Age group**	
−18–24 years	1% (11)
−25–29 years	5% (45)
−30–39 years	13% (134)
−40–49 years	16% (162)
−50–59 years	27% (265)
−60–69 years	22% (217)
−70+ years	16% (163)
Living alone without children	15% (246)
Employed/studying	62% (618)
**Past-year gambling**	
- Online casino	38% (381)
- Land-based casino	8% (81)
- Online horse betting	65% (646)
- Land-based horse betting	29% (291)
- Sports, live betting	48% (474)
- Sports, non-live betting	50% (495)
- Any sports betting	62% (619)
- Online poker	18% (178)
- Land-based poker	9% (87)
- Land-based electronic gambling machines	11% (113)
- Online bingo	22% (220)
**Ever felt need to seek problem gambling treatment**	
- Yes	5% (49)
- No	95% (945)
- Unsure/prefer not to answer	0% (3)
**Gambling severity**	
- No risk gambling	52% (514)
- Low-risk gambling	23% (230)
- Moderate-risk gambling	15% (154)
- Problem gambling	10% (99)

### Statistical Methods

Participants with and without a history of self-exclusion were compared using chi-square analyses. Among 17 cases with any missing data for psychological distress, four could be categorized as psychological distress as the available items summed up to a value of five or more, and three cases were categorized as non-psychological distress, as the sum was zero and only one item was missing. Variables with a statistically significant association (*p* < 0.05) with self-exclusion were entered simultaneously into a logistic regression analysis with self-exclusion as the dependent variable. In order to limit the number of variables entered into the model, moderate-risk/problem gambling (according to the PGSI) and perceived need for treatment seeking (both significantly associated with self-exclusion but also conceptually close to one another) were run against each other in a logistic regression, and here, moderate-risk/problem gambling was the strongest predictor, such that this variable was used in the overall regression model. In addition, within the smaller group of respondents having self-excluded, those with the longest time period chosen, and other respondents with self-exclusion, were compared with Fisher's exact test (as group sizes were small). Likewise, those reporting gambling during self-exclusion, and those who did not, were compared using the same method. Due to the low sample size in the specific comparisons within the group reporting self-exclusion, no regression analyses were carried out here.

## Results

Among 997 included individuals, six respondents (one percent) preferred not to answer the question about self-exclusion, whereas seven percent (*n* = 65, after correcting the option from the individual reporting a mistake) endorsed a history of self-exclusion, and 93 percent (*n* = 926) denied this. Among those having self-excluded, 57 percent (*n* = 37) were men, and 43 percent (*n* = 28) were women.

### Correlates of Self-Exclusion

Individuals reporting self-exclusion were significantly younger, more likely to be female, and more likely to score above cut-off for psychological distress, whereas they did not differ with respect to living alone without children or current employment/studying. Respondents who had self-excluded were more likely to have ever felt a need to seek problem gambling treatment, and more likely to screen positive for moderate-risk/problem gambling, and specifically they were more likely to belong to the subgroup with problem gambling. With respect to gambling types, respondents who reported self-exclusion were significantly more likely to report past-year gambling on online casino, land-based casino, online poker, electronic gambling machines, and online bingo, and less likely to report any sports betting, whereas they did not differ with respect to online horse betting or land-based horse betting (Table 2).

**Table 2:** Comparison of respondents with and without history of self-exclusion, chi-squared test (*N* = 991 after exclusion of six respondents with missing data for the self-exclusion item).

	**Individuals reporting self-exclusion (*n* = 65)**	**Individuals not reporting self-exclusion (*n* = 926)**	***p*-value**
Age			<0.001^a^
−18–24 years	2% (1)	1% (10)	
−25–29 years	8% (5)	4% (39)	
−30–39 years	29% (19)	12% (113)	
−40–49 years	11% (7)	17% (155)	
−50–59 years	29% (19)	26% (245)	
−60–69 years	14% (9)	22% (208)	
−70 years or older	8% (5)	17% (156)	
Female gender	43% (28)	24% (221)	<0.001
Psychological distress above cut-off	59% (38)	41% (373)	<0.01
Living alone without children	20% (13)	25% (231)	0.37
Employed/studying	69% (45)	61% (569)	0.21
Ever needed to seek treatment for gambling problems	22% (14)	4% (33)	<0.001
Moderate-risk/problem gambling	69% (45)	22% (202)	<0.001
-Problem gambling	40% (26)	7% (68)	<0.001
Past-year online casino gambling	89% (58)	34% (319)	<0.001
Past-year land-based casino gambling	18% (12)	7% (66)	0.001
Past-year online poker gambling	28% (18)	17% (157)	0.03
Past-year electronic gambling machines	23% (15)	10% (96)	0.01
Past-year online bingo	46% (30)	20% (188)	<0.001
Past-year sports betting (any)	43% (28)	63% (587)	0.001
Past-year online horse betting	53% (41)	65% (600)	0.78
Past-year land-based horse betting	22% (14)	30% (276)	0.16

a *Chi-square, linear-by-linear*.

In logistic regression, the reporting of self-exclusion remained significantly and positively associated with online casino gambling and level of gambling problems, and negatively associated with any sports betting, whereas age, gender, psychological distress and remaining gambling types did not remain significantly associated with self-exclusion (Table 3).

**Table 3:** Logistic regression of variables associated with self-exclusion (*N* = 981 after exclusion of respondents with any missing data for included variables).

	**Odds ratio**	**95-percent confidence interval**
Age	0.89	0.72–1.10
Male gender	1.20	0.63–2.29
Moderate-risk/problem gambling	3.99	2.08–7.64*
Online casino, past-year	8.02	3.34–19.29*
Land-based casino, past-year	1.39	0.58–3.33
Online poker, past-year	0.85	0.41–1.76
Land-based electronic gambling machines, past-year	0.92	0.42–2.03
Online bingo, past-year	1.40	0.76–2.60
Any sports betting, past-year	0.41	0.22–0.76*
Psychological distress	0.75	0.39–1.45

* *significant association with self-exclusion*.

### Self-Exclusion Time Periods

Among those reporting a self-exclusion history (*n* = 65), 23 percent (*n* = 15) reported having self-excluded for 1 month, 26 percent (*n* = 17) for 3 months, 22 percent (*n* = 14) for 6 months, 26 percent (*n* = 17) for at least 1 year, and three percent (*n* = 2) were uncertain or preferred not to report. Within the groups of individuals who had self-excluded, those reporting the longest time interval (*n* = 17) were not significantly different from others with respect to gender, age, employment, living conditions, psychological distress, or moderate-/risk or problem gambling. For gambling types, also, none reached a statistically significant association with self-excluding for the longest time interval, with the most marked differences in absolute numbers were for past-year online casino (76 percent of those reporting 1-year self-exclusion and 94 percent in other respondents reporting self-exclusion, *p* = 0.07, Fisher's exact test), electronic gambling machines (six vs. 29 percent, *p* = 0.09, Fisher's exact test), and any past-year sports betting (24 vs. 50 percent, *p* = 0.09, Fisher's exact test).

### Gambling Despite Self-Exclusion

Thirty-eight percent (*n* = 25) reported gambling during their self-exclusion, 58 percent (*n* = 38) denied this, and three percent (*n* = 2) preferred not to answer. Among the 25 individuals reporting such gambling despite being self-excluded, 52 percent (*n* = 13) reported gambling during self-exclusion on online casino, 16 percent (*n* = 4) online sports betting, 36 percent (*n* = 9) land-based lotteries, 21 percent (*n* = 3) online lotteries, four percent (*n* = 1) “restaurant” casino gambling, four percent (*n* = 1) land-based gambling in private homes, four percent (*n* = 1) for illegal gambling establishments, and 20 percent (*n* = 5) other games (three of them reported horse race betting). One of the latter individuals reported having self-excluded only from casino gambling and not from horse race betting.

The respondents endorsing the “gambling despite self-exclusion” item did not differ from those denying it with respect to gender (60 vs. 55 percent men, *p* = 0.71) or problem gambling status (80 vs. 61 percent moderate-risk/problem gambling, *p* = 0.10), whereas they were marginally more likely to report ever having felt a need to seek problem gambling treatment (33 vs. 13 percent, *p* = 0.06) and tended to be more likely to report psychological distress (72 vs. 49 percent, *p* = 0.07).

## Discussion

The present study reports on self-exclusion in a sample of online gamblers, and in the context of a novel, multi-operator, nationwide self-exclusion system, including the variables correlating with such self-exclusion. In addition, it further elaborates on the occurrence of gambling despite self-exclusion in this context. A history of self-excluding, reported by seven percent in this sample of past-year online gamblers, was clearly more common than the figures reported from the whole general population in Sweden, where around 50,000 individuals (i.e., well-below one percent of the adult population) are so far self-excluded (34). Importantly, respondents reporting self-exclusion were more likely to have gambling problems and markedly more common to be online casino gamblers, whereas the opposite was seen for sports betting. Gambling despite being self-excluded was common, with online casino being the most common gambling form in this group. In total, the study adds to the knowledge about characteristics of individuals who choose self-exclusion from gambling, a potential harm reduction tool in a condition where treatment seeking is known to be low (11). In particular, the study adds a perspective from online gamblers specifically, and in a type of broad, nationwide self-exclusion system rarely documented in the literature.

The fact that women were more likely to self-exclude may be in contrast to meta-analysis data on land-based self-exclusion, where a majority of those who had self-excluded were reported to be men (28). Motka and co-workers summarized gender in land-based and online self-exclusions programs, separately. In land-based programs, the percentage of men varied from 45 to 72 percent, compared to the 57 percent in the present study. In online-based self-exclusion services, likely more comparable to the present system, as many as 69–95 percent were men (27). Thus, while the *Spelpaus* system can likely be more precisely compared to previous online services, the lower proportion of men among those who had self-excluded in the present study may be considered to be in contrast to previous data. Also, the percentage of women in this sample of online gamblers was higher than in the official *Spelpaus* statistics in Sweden; thus, gender differences in this online sample appear to be smaller than in gamblers in general.

Altogether, this finding from the present study, as in a previous study in online gambling (33) points to a novel trend in online gambling in the present setting, where gender differences have become narrower (25) with increasing gambling problems in women (26), and that male gender may not even be not as clearly associated with gambling problems as before (33), and where female gender is associated with the gambling types most commonly reported in populations with problem gambling. In addition, it cannot be ruled out that self-exclusion may attract women and men differently and in different phases of life. Gambling is known to have a later age of onset in women, although problem gambling in women has been described to develop more rapidly after onset, often referred to as the telescoping phenomenon (41, 42). It remains to be studied in other research whether these trajectories from gambling onset to voluntary self-exclusion may differ with respect to gender. Here, although female gender was associated with self-exclusion, this association disappeared when controlling for online casino and other correlates.

Comparisons between those who reported self-exclusion and other gamblers demonstrated a relatively clear difference with respect to the past-year gambling types included; horse race betting and sports betting were not more common (and sports betting even significantly less common) among those who had self-excluded, whereas they were instead more likely to report the online and land-based chance-based games, as well as online poker. This picture is in line with the fact that online casino is the type and modality of gambling most commonly reported by gambling disorder patients seeking treatment in the present setting (24), and in line with the overall impression of online gambling as being more hazardous (23, 43). The negative association between sports betting and self-exclusion should be seen as relative with respect to other gambling types within the present study sample; all included subjects had a certain amount of online gambling, and therefore, the negative association with sports betting still does not exclude sports betting being a risk factor of self-exclusion in comparison to the full general population, but as a negative association in comparison to online casino gamblers in the sample.

Due to the relatively low absolute numbers of individuals who had self-excluded in the study, it was not possible to fully conclude whether there are characteristics separating individuals who choose a longer time period, i.e., the longest possible *Spelpaus* which can be breached only after 1 year, in comparison to those choosing a 1, 3, or 6-month exclusion. However, socio-demographic characteristics and psychological distress were not significantly different across the groups self-excluding for 1 year vs. shorter time, suggesting that more research is needed in order to better understand mechanisms behind choosing a longer or shorter self-exclusion period. No gambling patterns differed significantly between the groups, and the non-significant trends toward lower past-year gambling for some gambling forms in the 1-year exclusion group may primarily be interpreted as an effect of the theoretically lower gambling during a year when a person is self-excluded, particularly as the study was carried out after only around 17 months of this national self-exclusion system. It is beyond the scope of the present study to assess whether a longer time period of exclusion is more efficient than shorter periods, and reasons for choosing a longer or shorter self-exclusion period will need further study, and similar research needs to be repeated in different geographical settings with diverse gambling markets. Thus, further research is needed in order to highlight whether certain gambling patterns or other characteristics are likely to be associated with longer time periods chosen, and also, such future research may merit from studying self-exclusion systems in use for a longer time period, where, e.g., a 12-month exclusion period may also be preceded or followed by a longer period of non-exclusion than in the present relatively novel system.

It was expected that people with a history of self-exclusion had markedly more severe gambling habits, expressed both through the estimated gambling severity and through the item about perceived need for treatment. However, self-exclusion from gambling, particularly with the present system, may be chosen also by individuals who never gamble but who may want to feel safe from the risk of problem gambling, for example due to a previous gambling problem. Possibly, concerned significant others of individuals with problem gambling may potentially also decide to adhere to a non-gambling life-style and therefore to choose a self-exclusion in the absence of an own gambling problem. In addition, the present system makes direct marketing (such as through mail, e-mails or text messages) prohibited for operators to send to self-excluded individuals. As the present studied included past-year online gamblers with at least 10 occasions of such gambling, it does not give information about self-exclusion for such reasons. However, as participants were recruited from the general population due to their gambling practices, and not specifically due to a clinically diagnosed gambling problem, the present data may be a relatively good indicator of self-exclusion practices among online gamblers in this setting, regardless of the cause.

Interestingly, the significant difference in psychological distress between those reporting self-exclusion and other gamblers did not remain when controlling for other variables in the logistic regression analysis. Thus, for example due to the inclusion of problem gambling severity in the model, psychological distress did not demonstrate an association with self-exclusion over and above the difference explained by gambling patterns and other factors. However, it remains of interest to note that people who had ever chosen to self-exclude from gambling scored higher on psychological distress, again pointing to self-exclusion as a measure used to cope with problem gambling or as a harm reduction tool with or without formal treatment seeking. It remains to be studied, in other more in-depth study designs, whether specific mental health problems or psychological features may predict a willingness to self-exclude, and whether such mechanisms may remain even when controlling for the gambling pattern itself.

Gambling despite self-exclusion was relatively common in the group of gamblers reporting self-exclusion. Continued gambling despite self-exclusion has been shown to limit the effects of the intervention (44), and may seem particularly alarming given the severe consequences in an ongoing problematic gambling behavior, such as financial loss and severe mental health symptoms. There are likely no corresponding figures available for comparisons, as the present *Spelpaus* system involves all licensed gambling in the country, and therefore comparisons to more operator-specific or venue-based self-exclusions practices may be difficult. In the meta-analysis of self-exclusion interventions summarized by Kotter and co-workers, rates of “breaching” the self-exclusion (at the sites excluded from) ranged from 8 to 55 percent in exclusion systems of casinos, and 9 to 59 percent from exclusion systems from other land-based venues. As to the percentage gambling in other sites during self-exclusion, these figures ranged from 12 to 75 percent for casino self-exclusion programs, and from 23 to 59 percent for programs from other land-based venues (28). Although the programs summarized by Kotter and co-workers are all land-based, such that the comparison with the present study is difficult, a 38 percent rate of all-gambling breaching could be considered to be within the range of what can be expected from land-based self-exclusion systems. In studies assessing online self-exclusion systems, there is limited data of breaching patterns, while effects of short-term exclusion periods have been seen to be modest and in particular, self-exclusion may be less effective in individuals with the most pronounced gambling habits (45). Also, breaching self-exclusion on the present type of overall self-exclusion service involving major parts of the legal gambling market is previously undocumented, and analyses should be repeated in the present and other corresponding systems. Also, it merits further investigation whether such breaching involves illegal gambling or legal (but non-regulated in the own setting) offshore gambling operators which may theoretically involve higher risks and less of consumer protection compared to gambling occurring in the same context as available prevention and treatment tools. Likewise, it remains to be understood whether breaching self-exclusion in online gamblers can be seen as particularly hazardous or norm-breaking, given the fact that such gambling may occur in overseas sites beyond the regulatory systems of one's own setting.

In the study by McCormick and collaborators, self-exclusion violators were described not to differ substantially from those adhering to gambling abstinence; however, PGSI scores proved to be improved after a period of self-exclusion, although with less of a reduction in those breaching the exclusion (44). Although the field requires more research in different settings and across different self-exclusion program designs, it may be reasonable to hypothesize that individuals with problem gambling reporting continued gambling despite self-exclusion in the present study may represent a group corresponding to McCormick's and co-workers' description of the group improving partly but to a lesser extent than those not breaching the self-exclusion. In this sense, self-exclusion could indeed be seen as a harm reduction measure, i.e., a tool improving the clinical course although full abstinence is not achieved. While the present study is not an interventional or longitudinal study, such studies may be needed in order to further describe trajectories after exclusion from gambling.

The present study may have a number of implications for policy makers and for clinical settings, despite the relatively low absolute number of respondents with self-exclusion history and gambling despite self-exclusion. As this self-exclusion service is new, involving all licensed operators in a nationwide, authority-managed system hitherto not described, findings could be seen as preliminary and should both inform policy makers and suggest researchers to further studies in larger samples and with more in-depth study designs. However, from these findings so far, it can at least be concluded that even an official and nationwide self-exclusion from gambling does not rule out a risk of gambling to some extent during periods of self-exclusion, at least not in the sub-population of gamblers who have a relatively pronounced online gambling pattern as in the present study. Second, the risk of continued gambling, even though the study cannot establish the exact extent of such breaching of the self-exclusion, merits further research and potentially policy changes. Thus, screening for problem gambling, in mental health treatment settings, social services or by customer credit counselors, should continue to be emphasized even in the context of self-exclusion, as the latter cannot be assumed to provide a full protection against continued gambling. Third, the present study provides further data on the link between specific gambling types and gambling problems, in particular for online casino, which had by far the strongest association to a history of self-exclusion here, even when controlling for the gambling severity measure. Online casino gamblers demonstrated higher self-exclusion than sports bettors, even within this sample of online gamblers, a finding consistent with previous findings using the same methodology as here (33); rapid, chance-based games may be particularly problematic with respect to the risk of addictive behaviors, measured here through the choice to self-exclude. These issues are of importance to assess in future studies also with larger total samples and larger numbers of individuals having breached their self-exclusion, allowing for conclusions to be drawn with greater statistical power.

The present study has limitations, which are mainly related to the use of self-reported data, and because the actual temporal association between self-exclusion periods and gambling patterns, treatment needs or mental health could not be detected. The sample included depends on the population enrolled with an online web panel, and as shown in a previous study using the same methodology, this may include respondents with higher levels of income or education, than in the general population (33). Also, as the present study assessed online gamblers specifically, as the aim was to do so, conclusions cannot be drawn about how self-exclusion is used by gamblers who use exclusively land-based gambling types. Likewise, in addition to the present, first findings from a novel multi-operator self-exclusion service, further studies should provide more in-depth knowledge about gamblers' reasons for self-excluding with this particular type of system, and other qualitative aspects on how self-exclusion is perceived. While such study aims go beyond the ones of the present study, these aspects are likely to be of great relevance in order to optimize self-exclusion systems and increase their availability.

In addition, the study was carried out during the COVID-19 pandemic, and it is difficult to know whether that has an impact on data collection and findings in the study. Concerns have been raised about potential changes in gambling habits due to COVID-19, for example due to home confinement, time spent online, or lock-down of sports events (46), and these fears have led politicians to harm-reducing policy changes (although occurring in Sweden at a later date than the present data collection, 35). Theoretically, inclusion criteria, which referred to a gambling patterns on ten times or more during the past year, should not be severely affected by the COVID-19 and its impact on sports events. Within the present dataset, several land-based and sports-related gambling types could be suspected to be lower during the past 30 days than in a similar study carried out with the same inclusion criteria in 2018 (33), whereas online horse race betting appeared instead to be more common than in the comparison study from 2018. Likewise, individuals still reporting to gamble recently on the gambling types theoretically affected by the pandemic (i.e., those likely affected by lower attendance to land-based contexts and the short-term shortfall of sports events) appeared to have more gambling problems than other study respondents (31). Although COVID-19-related change in gambling has been reported to be modest (35), it cannot be excluded that the halted sports betting opportunities during the recruitment period may have influenced web panel members' perception of their own gambling habits. In addition, the study is conducted in only one country, and in a sample of active past-year online gamblers, such that rates of gambling problems in the whole study sample are naturally higher than in the general population, and generalizability to other countries or to populations of exclusively land-based gamblers may be limited.

Thus, while the potential impact of COVID-19 on study recruitment and past-30-day gambling reports is a limitation, this limitation should not be exaggerated, as the data reported in the study include any gambling on each specific gambling type, *either* during the past 30 days, *or* during the year prior to that. Also, a fully reliable sensitivity analysis, with respect to non-recent gambling in order to exclude the COVID-19-affected period, could not be conducted, as the data referring to the year prior to the most recent 30-day period was reported only for those denying each of the gambling types during the past 30 days. However, altogether, the choice to address each gambling type with a time frame ranging from either a very recent one, or a more longstanding one (far prior to the pandemic), should make the findings of the study more reliable. Also, it should be born in mind that the present study aimed to analyze gambling behaviors in online gamblers, defined with at least ten gambling sessions online such as on online casino or online betting. Thus, the gambling pattern for which they were included in the study was not primarily affected by the COVID-19 pandemic; although the content of the gambling during the most recent period may have changed, the possibility to gamble online was not technically affected by the pandemic, and consequences are likely more related to land-based gambling opportunities (31).

Also, an online survey necessarily limits the possibility to use longer or more extensive diagnostic tools, although problem gambling and psychological distress were measured using established tools. Another limitation, partly related to the necessarily brief format of a web survey, is that some further individual characteristics could not be investigated, such as a more thorough picture of the respondents' socio-demographic situation. For example, the present study in online gamblers did not address the geographical location, including the urbanicity or socio-economic situation of the respondents. Socio-economic situation is likely to affect the risk of problem gambling in general, as demonstrated in previous research (25, 47), including the geographical area of residence (47). Although it is less known whether this affects online gambling patterns as much as land-based gambling, more in-depth information about the living situation of the participants would have been of value. In addition, future research should assess similar broad self-exclusion systems after being in use for a longer time, as a person self-excluded a year prior to the study has been excluded from gambling for a large proportion of the time the system has been up and running, making it less likely for such a person to be included in the study. Still, however, the present study provides a broad picture of a relatively large sample of online gamblers, but future research may need to assess either larger samples or specifically recruited individuals with experience of self-exclusion.

In conclusion, assessments of multi-operator official self-exclusion systems are previously lacking, and the present study is therefore the first to elaborate of risk of breaching such a multi-operator self-exclusion. The present study concludes that online casino was strongly associated with a self-exclusion history, in contrast to sports betting, and that individuals with self-exclusion expectedly had higher degrees of gambling problems. The study also concludes that gambling despite self-exclusion, even in a broad nationwide multi-operator system, remains a challenge in online gamblers. Thus, while self-exclusion is a promising tool for prevention and harm reduction, more research is needed in order to evaluate and optimize its effects.

## Data Availability Statement

The datasets presented in this article are not readily available because Datasets are available after review by the ethics authority. Requests to access the datasets should be directed to anders_c.hakansson@med.lu.se.

## Ethics Statement

The studies involving human participants were reviewed and approved by Etikprövningsmyndigheten (Swedish Ethical Review Authority). The patients/participants provided their written informed consent to participate in this study.

## Author Contributions

AH and CW both contributed to the overall research idea. AH was the main responsible of the data collection, and wrote the first draft of the paper. CW made substantial contributions to the text of the manuscript and its overall design. Both authors approved the final version of the paper, results were interpreted, and discussed.

## Conflict of Interest

The research groups have overall research support from the state-owned gambling operator AB Svenska Spel, and from the regional hospital organization of southern Sweden (Region Skane).
